# Summary version of the Standards, Options and Recommendations for the use of analgesia for the treatment of nociceptive pain in adults with cancer (update 2002)

**DOI:** 10.1038/sj.bjc.6601086

**Published:** 2003-08-15

**Authors:** I Krakowski, S Theobald, L Balp, M P Bonnefoi, G Chvetzoff, O Collard, E Collin, M Couturier, T Delorme, R Duclos, A Eschalier, B Fergane, F Larue, M Magnet, C Minello, M L Navez, A Richard, B Richard, S Rostaing-Rigattieri, H Rousselot, N Santolaria, G Torloting, S Toussaint, N Vuillemin, J P Wagner, N Fabre

**Affiliations:** 1 Centre Alexis Vautrin, Vandoeuvre les Nancy, France; 2 Centre Paul Strauss, Strasbourg, France; 3 Centre hospitalier, Lons Le Saunier, France; 4 Centre Léon Bérard, Lyon, France; 5 Clinique Ste Clothilde, La Réunion, France; 6 Hôpital Salpétrière, Paris, France; 7 Centre Hospitalier Pierre Le Damany, Lannion, France; 8 Institut Curie, Paris, France; 9 Centre hospitalier, Le Mans, France; 10 Centre Hospitalier Universitaire-Faculté de médecine, Clermont Ferrand, France; 11 Hôpital Jean Minjoz, Besançon, France; 12 Centre hospitalier, Longjumeau, France; 13 Soin et Santé-HAD, Caluire, France; 14 Centre GF Leclerc, Dijon, France; 15 Hôpital de Bellevue, St Etienne, France; 16 Centre Hospitalier Universitaire, St Etienne, France; 17 Centre Hospitalier Universitaire Caremeau, Nîmes, France; 18 Hôpital St Antoine, Paris, France; 19 Centre de Moyen Séjour et Convalescence, Charleville Sous Bois, France; 20 Hôpital Gaston Doumergue, Nîmes, France; 21 Centre Hospitalier du Parc, Sarreguemines, France; 22 Centre hospitalier, Mulhouse, France; 23 Clinique de L'orangerie, Strasbourg, France; 24 FNCLCC, Paris, France

**Keywords:** pain, neoplasms, practice guideline

All patients have the right to expect to receive the best therapeutic means available to reduce their suffering. Recent evaluation of the French ministerial 3-year plan for the fight against pain by the French Society of Public Health (http://www.sfsp-france.org/Plan-Lutte-Douleur/Plan-Lutte-Douleur.htm) and observations of current practice show that the information and training of health professionals in this area should be continued. The necessity to fight against cancer pain has only recently become a priority. In 1996 a working group, set up by the French National Federation of Cancer Centres (Fédération Nationale des Centres de Lutte Contre le Cancer–FNCLCC), published clinical practice guidelines for pain management in adult and paediatric patients with cancer (Krakowski *et al*, 1996). These guidelines now require updating, but in this document only the pharmacological treatment of pain arising from excess nociception in adults with cancer is covered. The other sections of the original guideline are currently being updated in the context of a collaboration between the FNCLCC and the SETD (Society for the Study and Treatment of Pain (*Société d'Etude et de Traitement de la Douleur*) French section of the International Association for the Study of Pain (IASP) subgroup on cancer pain).

## OBJECTIVES

The main objective of this paper is to provide physicians with a reference document for the management of cancer pain in adults. Another objective is to provide guidance on the use of the different opioids now available since the number of drugs available has increased, as have drug formulations and modes of delivery. Another objective is also to remove all fears about the risk of addiction in patients with cancer.

The treatment of pain other than nociceptive pain (especially neuropathic pain), pain evaluation, the use of coanalgesics and pain management requiring specialist teams, will be covered in the updates currently being developed from the original document published in 1996 (Krakowski *et al*, 1996).

## METHODS

The section on ‘analgesic treatment’ in the document published in 1996 was examined by the working group to identify questions that required updating. These questions and the relevant key words were used to develop a search strategy that was used to search *Medline*®, and for particular questions, *Embase*® from January 1994 to March 1999, for relevant references, published in English or French. Additional references were provided by members of the working group.

After selection and critical appraisal of the articles, the working group proposed the ‘Standards’ and ‘Options’ for the management of nociceptive cancer pain in adult patients. Recommendations based on the best available evidence or expert agreement were developed using the SOR methodology (Fervers *et al*, 2001).

When all the members of the working group agree, based on the best available evidence, that a procedure or intervention is beneficial, inappropriate, or harmful, it is classified as a ‘*Standard*’, and when the majority agree, it is classified as an ‘*Option*’ (Table 1

**Table 1:** Definition of ‘Standards, Options and Recommendations’

*Standards*	Procedures or treatments that are considered to be of benefit, inappropriate or harmful by unanimous decision, based on the best available evidence
	
*Options*	Procedures or treatments that are to be considered of benefit, inappropriate or harmful by a majority, based on the best available evidence
	
*Recommendations*	Additional information to enable the available options to be ranked using explicit criteria (e.g., survival, toxicity) with an indication of the level of evidence

). In the SORs, there can be several ‘*Options*’ for a given clinical situation. ‘*Recommendations*’ provide additional information that enable the available options to be ranked using explicit criteria (e.g. survival, toxicity) with an indication of the level of evidence. These recommendations help clinicians to select an appropriate option. Thus, clinicians can make choices for the management of patients using this information and taking into consideration local circumstances, skills, equipment, resources and/or patient preferences. The adaptation of the SOR to the local situation is allowable if the reason for the choice is sufficiently transparent and this is crucial for successful implementation. Inclusion of patients in clinical trials is an appropriate form of patient management in oncology and is recommended frequently within the SORs, particularly in situations where only weak evidence exists to support a procedure or an intervention.

The type of evidence underlying any ‘*Standard*’, ‘*Option*’ or ‘*Recommendation*’ is indicated using a classification developed by the FNCLCC based on previously published methods. The level of evidence depends not only on the type and quality of the studies reviewed, but also on the concordance of the results (Table 2

**Table 2:** Definition of level of evidence

*Level A*
There exists a high standard meta-analysis or several high standard randomised clinical trials that give consistent results

*Level B*
There exist good quality evidence from randomised trials (B1) or prospective or retrospective studies (B2). The results are consistent when considered together

*Level C*
The methodology of the available studies is weak or their results are not consistent when considered together

*Level D*
Either the scientific data do not exist or there is only a series of cases

*Expert agreement*
The data do not exist for the method concerned, but the experts are unanimous in their judgement

). When no clear scientific evidence exists, judgement is made according to the professional experience and consensus of the expert group (‘expert agreement’), but this is then validated by the peer-review process. For this update, only a few randomised clinical trials have been identified, and their conclusions are generally weak. Thus, much of the information in this document represents the ‘state of the art’ on this subject in France and is supported by expert agreement. Much of the information in this document is based on the World Health Organisation (WHO) guidelines initially published in 1986 (World Health Organisation, 1986).

The integral version of this document was peer reviewed by independent experts, and their comments were taken into consideration in the preparation of the final document. The SOR guidelines are considered to be validated when the members of the working group give their agreement for publication. This integral version is available on the FNCLCC web site (http://www.fnclcc.fr). A summary version was prepared in French (Krakowski *et al*, 2003) based on the full text version, and this is the English version of that summary.

### General principles

Analgesic treatment should be constantly adapted to the clinical situation in which it is used (standard, expert agreement). In addition to the criteria used to choose the appropriate treatment (patient age, performance status, past history, pain aetiology, potential side effects, etc.), an understanding of the physiopathological mechanisms of the pain is essential (standard, expert agreement).

**Figure 1:**
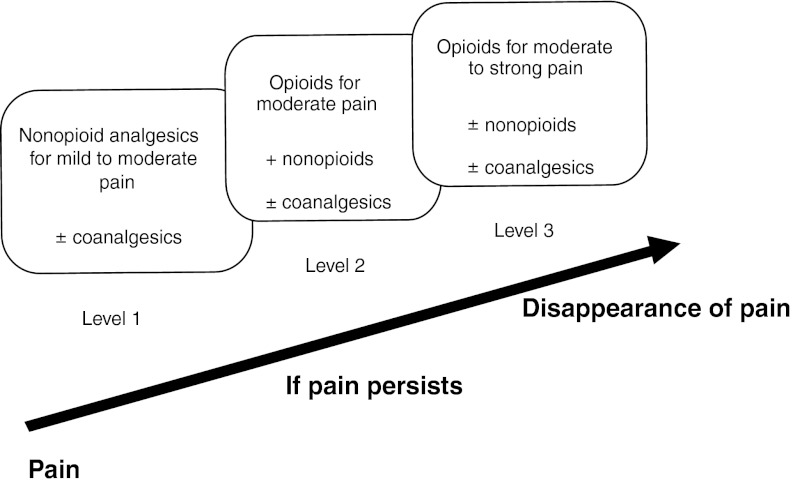
Modified WHO analgesic ladder.

The WHO analgesic ladder is used to classify three levels of pain as summarised in Figure 1

. The strategy originally developed by WHO for the management of nociceptive pain in patients with cancer is based around five essential principles and these remain generally relevant today (standard, expert agreement):

- oral drug administration;
- drug administration at regular intervals;
- drug administration conforming to the three-step WHO ladder (Figure 1);
- personalised administration;
- constant attention to detail.

The planned treatment should be written down and explained to the patient. It should anticipate breakthrough pain and side effects. It should be re-evaluated regularly (standard, expert agreement).

The timing of progression from one step of the therapeutic ladder to the next is dependent on the duration of action of the analgesic and the intensity of the pain (standard, expert agreement).

Two products of the same pharmacological class, with the same kinetics (e.g., two sustained-release opioids), should not be prescribed together (standard, expert agreement).

Coanalgesics can be used concurrently at each level in the WHO analgesic ladder (standard, expert agreement).

The prescription of strong opioids as first-line analgesia should be considered in patients with very severe pain (option, expert agreement).

### Nonopioid analgesics (WHO level 1)

Nonopioid analgesics should be used for mild to moderate pain (standard, expert agreement). Nonopioid analgesics (WHO level 1) can be combined with opioid analgesics (WHO levels 2 and 3) (option, expert agreement).

Acetaminophen (1 g every 4–6 h) is recommended as first-line treatment for mild to moderate pain (recommendation, expert agreement). The maximum dose in the French product licence is 4 g/day. Acetaminophen can cause hepatic toxicity if the daily recommended dose is exceeded. This justifies cautious use in patients with liver failure (recommendation, expert agreement).

The use of nonsteroidal anti-inflammatory drugs (NSAID) is recommended for treating inflammatory pain, particularly bone pain (recommendation, expert agreement). NSAIDs should not be used with methotrexate (standard, expert agreement). When NSAIDs are prescribed, the potential risk when used in conjunction with nephrotoxic (particularly cisplatin) or myelotoxic chemotherapy should not be ignored (standard, expert agreement). If gastrointestinal symptoms develop in patients taking NSAIDs, the necessity to use an NSAID at all and/or the need for gastroscopy and/or the prescription of a proton pump inhibitor should be reviewed (standard, expert agreement).

COX2 inhibitors have not been evaluated in clinical trials of patients suffering from cancer pain and they are not licensed for use in cancer patients in France. Nefopam and floctafenin are not indicated as first-line treatments for chronic cancer pain (recommendation, expert agreement).

Dipyrone is not advised, except in specific situations, because of the serious, unpredictable side effects that have been reported (recommendation, expert agreement).

### Classification of the opioids

Based on their analgesic efficacy, opioids are classed as ‘weak’ for moderate pain (WHO level 2 — classified as prescription drugs in France) and ‘strong’ for moderate to severe pain (WHO level 3 — classified as special prescription drugs in France except for buprenorphine and nalbuphine (prescription drug).

Opioids can be classed into three categories based on the type of action they have on receptors: pure agonist, partial agonist–antagonist or mixed agonist–antagonist. Drugs in different categories should not be prescribed at the same time (standard, expert agreement).

### ‘Weak’ opioid analgesics (WHO level 2)

‘Weak’ opioid analgesics should be used in patients with moderate pain (standard, expert agreement).

Weak opioid analgesics can be used alone or in combination with a level 1 analgesic (option, expert agreement). The following products can be used: codeine, dextropropoxyphene, dihydrocodeine, tramadol (option, expert agreement). There are no absolute criteria to guide the selection of the different drugs.

Tramadol should not be combined with monoamine oxidase inhibitors (standard, expert agreement). Tramadol should be used with caution in patients with a risk of epilepsy and when used in combination with antidepressants (recommendation, expert agreement).

Dextropropoxyphene should not be used in combination with carbamazepine as it will induce an increase in the plasma concentration of carbamazepine (recommendation, expert agreement).

Constipation should always be anticipated in patients receiving codeine (standard, expert agreement).

### Strong opioid analgesics (WHO level 3)

Strong opioid analgesics should be used in patients with moderate to severe pain (standard, expert agreement).

#### Morphine

Except in specific situations, oral morphine is the first-line WHO level 3 opioid of choice (standard, expert agreement). Oral morphine should be given without delay to patients whose pain is uncontrolled by step 1 and 2 treatments (standard, expert agreement).

Morphine should be prescribed in an oral form, either as tablets or capsules of immediate-release morphine sulphate, as tablets or capsules of sustained-release morphine sulphate, or as morphine hydrochloride solution (standard, expert agreement).

In all patients treated previously with another strong opioid, the starting dose for morphine should be calculated using equianalgesic dose ratios (standard, expert agreement).

In patients receiving baseline treatment with opioids, an immediate-release formulation must be prescribed concurrently for the treatment of breakthrough or incident pain (standard, expert agreement).

It is advisable to be cautious by prescribing doses at the lower end of the equianalgesic dose range and providing rescue doses as needed, rather than immediately subjecting the patients to doses at the upper limit of the range (standard, expert agreement).

When oral administration is impossible, the preferred routes are transdermal (e.g. fentanyl), or continuous parenteral administration with patient-controlled analgesia (e.g. morphine) (option, expert agreement). The choice of dose should take into consideration equianalgesic dose ratios (recommendation, expert agreement).

The other routes of administration for morphine and the other opioids are rarely indicated. They should be used taking into consideration the benefit to risk ratio, the training of the personnel involved (including close family and friends), and the constraints of the follow-up, particularly for patients at home (recommendation, expert agreement).

Treatment with opioids (particularly oral morphine) should never be stopped abruptly (standard, expert agreement). No specific protocol for reducing the dose of opioids has been validated. Dose reduction should be in steps of 30–50% over about a week, depending on the clinical situation (reappearance of pain, development of withdrawal symptoms, etc.) (recommendation, expert agreement).

#### Other opioids

Although buprenorphine is used in some countries, it cannot be recommended as a WHO level 3 opioid since other opioids have become available (recommendation, expert agreement).

The use of fentanyl patches at 25 *μ*g h^−1^ is one therapeutic option for the initiation of opioid treatment in patients with stable pain. This is appropriate for patients who do not need frequent breakthrough doses and do not have intense pain that requires parenteral dosing because of the faster onset of action. It can be useful in the following situations (option, expert agreement):

- oral administration impossible because of uncontrolled nausea and vomiting;
- risk of bowel obstruction;
- poor digestive absorption: fistula; short or irradiated small intestine; gastrointestinal damage following surgery; severe diarrhoea; etc.;
- moderate chronic renal failure (fentanyl is mainly metabolised through a hepatic route);
- to reduce the number of tablets in patients distressed by a large number of medications.

Hydromorphone is indicated for patients with severe cancer pain when there is resistance or intolerance to morphine (option, expert agreement).

Oxycodone is another alternative to oral morphine for the treatment of severe cancer pain when there is resistance or intolerance to morphine (option, expert agreement).

Pethidine has no place as a WHO level 3 opioid since other opioids have become available (option, expert agreement).

Transmucosal fentanyl should only be used to treat breakthrough pain as a complement to baseline opioid analgesia in patients with chronic cancer pain. The high cost of this treatment should be taken into consideration (option, expert agreement).

#### Titration

The initial and subsequent dose titration (dose readjustment) of WHO level 3 opioids can be undertaken with a sustained-release preparation in combination with an immediate-release form, or with an immediate-release form only, particularly in ‘frail’ patients (option, expert agreement).

At the time of initial titration, at least once-daily self-assessment (particularly for patients at home) or assessment by a health professional is necessary to judge the analgesic efficacy and to detect any side effects (standard, expert agreement).

There is no upper limit to the dose of a pure agonist opioid as long as the side effects can be controlled (standard, expert agreement).

Rescue doses should be calculated based on the daily opioid dose (standard, expert agreement).

Each immediate-release opioid rescue dose should correspond to 10% of the daily dose of sustained-release opioid (recommendation, expert agreement).

Patients who have uncontrolled pain can take a rescue dose every hour for up to 4 h, before consulting a physician. If pain relief is not obtained after the four consecutive doses, the patient should be reassessed, in a hospital if necessary (recommendation, expert agreement).

#### Prescription

Outside the hospital setting, prescriptions for opioids should be written out in full on security prescription forms. All oral forms of morphine and most opioids can be prescribed for a maximum of 28 days. For injectable forms, the prescription is restricted to 7 days or to 28 days when used in an infusion system.

In hospital, it is not obligatory to write the prescription on a security prescription form. In practice, the procedure will vary depending on local practice.

The loss or theft of a security prescription form should be declared. In France, this declaration should be made to the departmental medical supervisory council, the regional pharmacy inspectorate as well as to the local police.

### Side effects of oral morphine

All opioids have similar side effects. The ‘Standards, Options, and Recommendations’ below relate to oral morphine.

There is wide inter- and intrapatient variation in the occurrence of side effects. The occurrence of side effects is not necessarily related to an overdose. Miosis is a sign of morphine usage, but it is not a sign of overdosage. The risk of overdose is low in patients with cancer pain, when they are followed-up and evaluated regularly and when they receive morphine continuously for a long period. Psychological dependence is rare in patients with cancer.

Tolerance and physical dependence are not problems in patients treated with oral morphine for cancer pain. The prescription should be uninterrupted. The coprescription of an opioid receptor agonist and an antagonist should be avoided to prevent physical dependence. Drowsiness occurs principally during the treatment titration phase and usually disappears within a few days. In patients with persistent or recurrent drowsiness, the presence of metabolic disorders or potentiation by other drugs should be considered (standard, expert agreement). However, if the morphine is found to be the cause, its dose should be reduced or the drug changed (rotation) (option, expert agreement). Dose reduction should be preferred if the pain is well controlled (recommendation, expert agreement).

With the exception of constipation, other side effects tend to disappear in the first few days or weeks of treatment with oral morphine. The patient should be informed about the possibility of side effects, particularly the most frequent: constipation, nausea, drowsiness (standard, expert agreement).

A laxative, to prevent constipation, should be prescribed during the treatment period and this should be associated with advice on diet and hydration (standard, expert agreement).

In patients with nausea and vomiting during treatment, other possible causes should be excluded first. If this persists, an antiemetic should be prescribed for a few days (standard, expert agreement).

There is no contraindication for the prescription of opioids in patients with asthma and/or respiratory failure (standard, expert agreement).

In patients receiving oral morphine who have resistant side effects, the options are either to change the administration route or to change the opioid (rotation, see below):

- in the presence of very unstable, intense pain, preference should be given to intravenous or subcutaneous patient-controlled analgesia (option, expert agreement);
- in other situations (stable and/or moderate pain), rotation and/or intravenous or subcutaneous patient-controlled analgesia are possibilities (option, expert agreement).

### Opioid rotation

Opioid rotation is defined as the substitution of one opioid by another and is justified if this results in an improved benefit to risk ratio. The main indication for opioid rotation is the occurrence of resistant side effects (particularly disorders of cognitive function, hallucination, myoclonus and nausea), despite adequate symptomatic treatment (usually in patients receiving high doses of opioids) (standard, expert agreement).

Another indication for opioid rotation is the rare occurrence of resistance to opioids, defined not only by an absence of efficacy, but also by an absence of side effects despite a rapid increase in opioid dose (standard, expert agreement).

Opioid rotation is possible between all the pure agonists: morphine, fentanyl, hydromorphone, oxycodone (option, expert agreement).

There are no validated criteria to guide drug selection when undertaking an opioid rotation, apart from the individual precautions for use of each drug and the relative contraindications for each individual opioid (recommendation, expert agreement).

The occurrence of side effects in patients in whom the dose is increased does not always necessitate opioid rotation (recommendation, expert agreement).

Opioid rotation should take into consideration equianalgesic doses, but it is always advisable to favour safety over rapid action by using the lowest value of the conversion range (recommendation, expert agreement).

### Precautions for use, compatibility, drug combinations and opioids

When administering opioids parenterally, the physicochemical compatibility with all other drugs needs to be taken into consideration, as well as the risk of side effects arising from the combination (standard, expert agreement).

In the event of metabolic failure, particularly liver or renal, the same precautions should be taken for WHO level 2 and 3 opioids (standard, expert agreement).

In the event of metabolic failure, opioids should be prescribed with caution taking the following into account (recommendation, expert agreement):

- selection of opioids taking into consideration their preferential metabolic pathway and active metabolites;
- administration of oral or parenteral immediate-release forms;
- administration of lower doses (much lower doses may be appropriate depending on the extent of the metabolic failure);
- cautious titration taking into consideration the efficacy and duration of action of the first dose to calculate the subsequent doses and intervals. After a few days of a stable dose, it may be possible to consider using a sustained-release formulation with adjustment, if necessary, of the rescue dose.

Rapid or immediate release formulations should be used initially in patients with a short or irradiated small intestine (recommendation, expert agreement). In elderly patients, lower doses and/or longer intervals between the doses is recommended. Dose titration, following the above rules, will facilitate pain control (recommendation, expert agreement).

### Actions to be taken in the event of opioid overdosage

Overdosage with oral morphine, and with opioids in general, is characterised mainly by increasing drowsiness. This is accompanied by respiratory failure, seen as respiratory depression and an increased expiratory pause (risk of apnoea).

Treatment of severe respiratory depression (respiratory frequency less than about 8 min^−1^) principally involves stopping opioid treatment, stimulating the patient, oxygen therapy, and the injection of an opioid-antagonist (naloxone). Constant surveillance is required. Transfer to a medical intensive care unit may be necessary, particularly if the patient is at home (standard, expert agreement).

In the absence of a published, validated administration protocol for naloxone, the following protocol is recommended (recommendation, expert agreement):

- prepare an ampoule of 1 ml (0.4 mg) diluted to 10 ml with a 5% saline or glucose solution;
- give an intravenous injection of 1 ml every 2 min until the respiratory frequency increases to 10 min^−1^. This titration aims to eliminate the respiratory depression, but not the analgesia;
- infuse two ampoules diluted in 250 ml over 3–4 h, repeat if necessary, depending on the respiratory frequency and taking into consideration the elimination time of the drug responsible for the overdosage.

In other situations of overdosage, a ‘therapeutic window’, adapted to the drug's half-life and the intensity of the symptoms, should be identified (standard, expert agreement).

If in the slightest doubt, the patient should be referred to an anaesthetist for supervision of treatment and follow-up (standard, expert agreement).

## Appendix

### Reviewers

JP Alibeu (Centre Hospitalier Universitaire, Grenoble), A Aubrege (Villers lès Nancy), E Bisot (Coullons), F Boureau (Hôpital St Antoine, Paris), L Brasseur (Hôpital Amboise Paré, Boulogne), B Burucoa (Hôpital St André, Bordeaux), JB Caillet (Hôpital Louis Pradel, Lyon), L Chassignol (Centre hospitalier, Privas), F Chast (Hôpital Hôtel Dieu, Paris), P Clerc (Issy les Moulineaux), P Colombat (Centre Hospitalier Universitaire, Tours), R Coulouma (Centre Val d'Aurelle, Montpellier), D d'Herouville (Association François-Xavier Bagnoud, Paris), M Di Palma (Institut Gustave Roussy, Villejuif), S Donnadieu (Hôpital Georges Pompidou, Paris), C Dormard (Saclay), G Ducable (Centre Hospitalier Universitaire Charles Nicole, Rouen), M Durand (Institut Bergonié, Bordeaux), P Fargeot (Centre Georges François Leclerc, Dijon), L Feuvret (Centre Médico-chirurgical, Chaumont), JC Fondras (Centre Hospitalier, Bourges), JM Gomas (Centre hospitalier Ste Perrine, Paris), G Ganem (Centre Jean Bernard, Le Mans), MT Gatt (Hôpital de Avicenne, Bobigny), F Hirszowski (Paris), B Hoerni (Institut Bergonié, Bordeaux), SD Kipman (Paris), A Margot-Duclot (Fondation Rothchild, Paris), J Meynadier (Centre Oscar Lambret, Lille), A Millet (Tarcenay), S Monin (Hôpital Necker, Paris), A Muller (Hôpital Civil, Strasbourg), J Peny (Centre François Baclesse, Caen), D Roy (Clinique Mutualité de la Sagesse, Rennes), G Simmonet (Centre Hospitalier Universitaire, Bordeaux), M Sindou (Hôpital Neurologique, Lyon), D Sommelet (Centre Hospitalier Universitaire, Vandoeuvre-lés-Nancy), A Suc (Centre Hospitalier Universitaire Purpan, Toulouse).
